# A structural pathway from wage discrepancy to turnover intention among hospital nurses: the mediating roles of effort–reward imbalance, organizational justice, burnout, and organizational commitment

**DOI:** 10.3389/fpubh.2026.1966344

**Published:** 2026-10-20

**Authors:** Mi Yu

**Affiliations:** College of Nursing, Sustainable Health Research Institute, Gyeongsang National University, Jinju, Republic of Korea

**Keywords:** burnout, effort–reward imbalance, nurses, organizational commitment, organizational justice, turnover intention, wage discrepancy

## Abstract

**Background:**

Perceived wage inadequacy is an important concern for nurse retention, but the mechanisms through which perceived wage discrepancy is associated with turnover intention remain unclear. Thus, this study aimed to examine the structural pathways linking wage discrepancy to turnover intention among hospital nurses, focusing on the roles of effort–reward imbalance, organizational justice, burnout, and organizational commitment.

**Methods:**

This cross-sectional study was conducted with 792 nurses employed in tertiary hospitals, general hospitals, and hospitals across South Korea. Wage discrepancy was calculated as the ratio of perceived appropriate annual wage to current annual wage. Structural equation modeling was used to examine the hypothesized relationships and direct, indirect, and total effects among the study variables.

**Results:**

The mean wage discrepancy ratio was 1.14, indicating that nurses perceived an appropriate wage to be approximately 14% higher than their current wage. Greater wage discrepancy was positively associated with effort–reward imbalance (*β* = 0.303, *p* < 0.001) and negatively associated with organizational justice (*β* = −0.281, *p* < 0.001). Effort–reward imbalance was positively associated with burnout, whereas organizational justice was negatively associated with burnout and positively associated with organizational commitment. Burnout showed the strongest direct association with turnover intention (*β* = 0.667, *p* < 0.001), whereas organizational commitment was negatively associated with turnover intention (*β* = −0.245, *p* < 0.001). Wage discrepancy showed no significant direct association with turnover intention but had a significant indirect association through the proposed pathways (*β* = 0.226, *p* = 0.001). The model accounted for 76.2% of the variance in turnover intention in the present sample.

**Conclusion:**

Wage discrepancy is associated with nurses’ turnover intention primarily through organizational and psychological pathways rather than through a direct effect. These findings suggest that the association between compensation and turnover intention may depend not only on wage level but also on how nurses perceive the adequacy and fairness of rewards relative to their contributions. Therefore, nurse retention strategies should address compensation adequacy and fairness while reducing burnout and strengthening organizational commitment.

## 1 Introduction

Geographic maldistribution of healthcare professionals and workforce imbalances across healthcare institutions are important issues affecting the stability of healthcare systems and the quality of healthcare services. In particular, early turnover among nurses and their movement between healthcare institutions can result in recurrent staffing shortages, increase both the workload of remaining nurses and the organizational costs associated with recruiting and training new staff, and adversely affect continuity of nursing care and patient safety through the loss of experienced nurses (1, 2). Therefore, nurse turnover should be understood not only as an issue of individual career choice or organizational adjustment but also as a phenomenon arising from the interaction of labor market structures, compensation systems within healthcare institutions, and organizational environments (2).

In Korea, despite the continued expansion of the nursing workforce supply, disparities in the distribution of nurses across healthcare institution types and geographical regions persist (3, 4). In particular, small- and medium-sized hospitals and healthcare institutions in nonmetropolitan areas experience greater difficulties in recruiting and retaining nurses (3, 4), and such workforce imbalances may adversely affect the quality of healthcare services (5). Therefore, identifying the factors associated with nurses’ turnover and retention is an important step toward securing a stable nursing workforce and reducing regional disparities in nurse distribution (1).

Wages and compensation are among the major factors influencing the mobility and distribution of the nursing workforce. In Korea, nurses’ wages vary substantially according to healthcare institution type, hospital size, geographical location, and clinical experience (6, 7), and these wage differentials may influence nurses’ choice of healthcare institution and job mobility. In particular, because larger healthcare institutions generally provide higher wages and greater opportunities for career development than smaller institutions, wage differentials across healthcare institutions may serve as an economic incentive for nurses to move to larger hospitals or metropolitan areas (3, 4, 6). Thus, nurses’ wages need to be considered not only as individual compensation but also in relation to disparities in the distribution of the nursing workforce across healthcare institutions and geographical regions.

Nurses’ wages and compensation are also important policy issues for nursing workforce recruitment and retention. According to the Organization for Economic Co-operation and Development (OECD), hospital nurses across OECD countries earned, on average, approximately 20% more than the average wage of all employees in 2023, although substantial differences existed across countries. These differences in remuneration may also influence the international mobility of nurses (8). Importantly, nurses’ compensation extends beyond remuneration for labor and may also reflect organizational and societal recognition of their professional responsibilities and workload and is, therefore, highly relevant to nursing workforce recruitment and retention.

Studies from diverse national contexts, including South Korea, China, Saudi Arabia, and Sweden, together with systematic reviews and meta-analyses across countries, have consistently identified wages and compensation as important factors associated with nurse turnover and retention. Higher wages are generally associated with lower turnover intention and actual turnover, whereas low wages, dissatisfaction with compensation, and perceptions of unfair compensation are associated with greater turnover intention (9–13). Collectively, these findings indicate that compensation is a consistent concern for nurse retention across different national and healthcare contexts, although the mechanisms through which perceived wage inadequacy translates into turnover intention remain unclear.

However, the influence of wages on turnover may not be fully explained by absolute wage levels alone. Even when nurses receive the same wage, their responses may differ depending on whether they perceive the compensation as appropriate relative to their workload, responsibilities, experiences, and contributions. Therefore, wage discrepancy, defined as the difference between current wage and the wage perceived by nurses as appropriate, may more directly reflect an individual’s evaluation of the adequacy of compensation. A greater wage discrepancy may be associated with perceptions that compensation is insufficient relative to effort or that the organization’s reward system is unfair and, in turn, with greater turnover intention.

This relationship can be explained by effort–reward imbalance (ERI) theory, which proposes that a lack of reciprocity occurs when employees perceive that the rewards they receive, including wages, recognition, job security, and career opportunities, are insufficient relative to the effort they invest in their work (14). Persistent imbalance between effort and reward is associated with psychological stress and adverse work-related outcomes. Particularly in nursing environments characterized by high job demands and responsibilities, this imbalance may be associated with depletion of physical and emotional resources. Accordingly, the greater the discrepancy between current and perceived appropriate wages, the more likely nurses are to perceive their rewards as insufficient relative to their efforts and consequently experience greater ERI.

Wage discrepancy may also be associated with perceptions of organizational justice. Organizational justice refers to the extent to which employees perceive the distribution of rewards, decision-making procedures, and interpersonal treatment within an organization as fair (15). When nurses perceive their wages as inadequate relative to their workload, responsibilities, experiences, and contributions to the organization, they may perceive not only the outcomes of reward allocation but also the criteria and procedures used to determine those rewards as unfair. Conversely, perceptions of fairness in compensation systems and decision-making processes may be associated with greater trust in and psychological attachment to the organization.

In turn, ERI and low organizational justice may be associated with burnout. When nurses continuously exert high levels of effort without receiving commensurate rewards or perceive the organization’s compensation system as unfair, their psychological resources may become depleted. Greater burnout may, in turn, be associated with higher turnover intention as nurses consider leaving their current organization to escape stressful working conditions (15, 16).

Organizational commitment is another important organizational attitude in this process. When nurses perceive compensation and decision-making processes as fair, their trust in and sense of belonging to the organization may be strengthened, which may be associated with greater organizational commitment. In contrast, burnout may be associated with weaker psychological attachment to the organization and lower organizational commitment. Subsequently, lower organizational commitment may be associated with lower willingness among nurses to remain in their current organization and with greater turnover intention (16, 17).

Taken together, previous studies have documented associations among wages, ERI, organizational justice, burnout, organizational commitment, and turnover intention. However, these relationships have largely been examined separately, and relatively little is known about how they are linked within an integrated process that connects wage discrepancy with turnover intention. In particular, evidence examining both the direct and indirect pathways from wage discrepancy to turnover intention through ERI, organizational justice, burnout, and organizational commitment remains limited among nurses working in healthcare institutions across Korea.

The present study addresses this gap by integrating these constructs within a single structural model to examine how wage discrepancy is associated with turnover intention through psychological and organizational pathways. Accordingly, this study aimed to examine the structural relationships among wage discrepancy, ERI, organizational justice, burnout, organizational commitment, and turnover intention. Specifically, we examined the direct association between wage discrepancy and turnover intention and its indirect associations through ERI, organizational justice, burnout, and organizational commitment.

The purposes of this study were as follows:

1. To examine the levels and characteristics of annual wage, wage discrepancy, ERI, organizational justice, burnout, organizational commitment, and turnover intention among nurses working in healthcare institutions nationwide.
2. To identify differences in annual wage according to hospital type and geographical location.
3. To test a structural equation model explaining nurses’ turnover intention based on wage discrepancy, ERI, organizational justice, burnout, and organizational commitment.
4. To examine the direct, indirect, and total effects among the study variables and compare the hypothesized model with competing models to identify the most theoretically and empirically appropriate structural model.

## 2 Conceptual framework

### 2.1 Wage discrepancy as a structural antecedent of turnover intention

This study drew on effort–reward imbalance (ERI) theory and organizational justice theory as complementary conceptual perspectives for understanding the relationship between wage discrepancy and turnover intention. ERI theory explains that psychological stress and negative organizational outcomes occur when employees do not receive sufficient rewards, such as pay, recognition, job security, and career development opportunities, relative to the effort they invest in their work (14). In contrast, organizational justice theory posits that organizational members’ perceptions of the fairness of reward distribution, decision-making procedures, and interactions within the organization have important effects on organizational attitudes and behaviors (15). Thus, the two perspectives represent conceptually distinct evaluations of compensation: ERI focuses on reciprocity between effort and reward, whereas organizational justice focuses on the fairness of organizational processes and outcomes.

In this study, wage discrepancy was conceptualized as the difference between nurses’ current annual wage and perceived appropriate annual wage. A greater discrepancy between current and perceived appropriate annual wages may be associated with stronger perceptions that the rewards received are insufficient relative to the effort invested and that the organization’s reward allocation and decision-making processes are less fair. Previous studies have shown that higher pay is associated with lower turnover intention (12), whereas current wages and hospital size are associated with nurses’ actual turnover (11). However, the direct association of pay with turnover intention may be attenuated after accounting for job-related and organizational factors (18), suggesting that compensation may be linked to turnover intention through psychological and organizational processes. Accordingly, wage discrepancy was positioned as an antecedent of ERI and organizational justice in the proposed model.

### 2.2 Mediating mechanisms linking wage discrepancy and turnover intention

ERI and organizational justice were assumed to be key mediating mechanisms linking wage discrepancy with turnover intention. When nurses perceive that they continuously exert high levels of effort without receiving corresponding rewards or regard the organizational compensation system as unfair, their psychological resources may become depleted, which may be associated with greater burnout. ERI has been significantly associated with nurse burnout (19), and a study of emergency nurses also identified an association between ERI and emotional exhaustion (20). Organizational justice has also been associated with lower burnout (21).

Meanwhile, organizational justice and burnout were subsequently hypothesized to be associated with organizational commitment. When organizational compensation systems and decision-making processes are perceived as fair, trust in the organization and a sense of belonging may be strengthened and associated with greater organizational commitment (22, 23). In contrast, burnout may weaken psychological attachment to the organization and be associated with lower organizational commitment. Ultimately, burnout is positively associated with turnover intention (15), whereas organizational commitment is a key organizational attitude that is negatively associated with turnover intention (17). Therefore, this study hypothesized that ERI and organizational justice would be associated with turnover intention through burnout and organizational commitment, thereby mediating the relationship between wage discrepancy and turnover intention.

### 2.3 Hypothesized structural model

Based on the theories and previous studies described above, wage discrepancy was specified as the exogenous variable, with ERI and organizational justice positioned as first-stage intervening variables. Burnout was subsequently specified as a psychological response, organizational commitment as an organizational attitude, and turnover intention as the final outcome variable. Specifically, greater wage discrepancy was hypothesized to be associated with higher ERI and lower organizational justice, which, in turn, were expected to be associated with greater burnout and lower organizational commitment. Burnout and organizational commitment were subsequently hypothesized to be associated with turnover intention. The structural model included both a direct path from wage discrepancy to turnover intention and indirect pathways through ERI, organizational justice, burnout, and organizational commitment (Figure 1). Accordingly, the proposed model was designed to examine whether wage discrepancy was associated with turnover intention directly or primarily through these psychological and organizational pathways.

**Figure 1:**
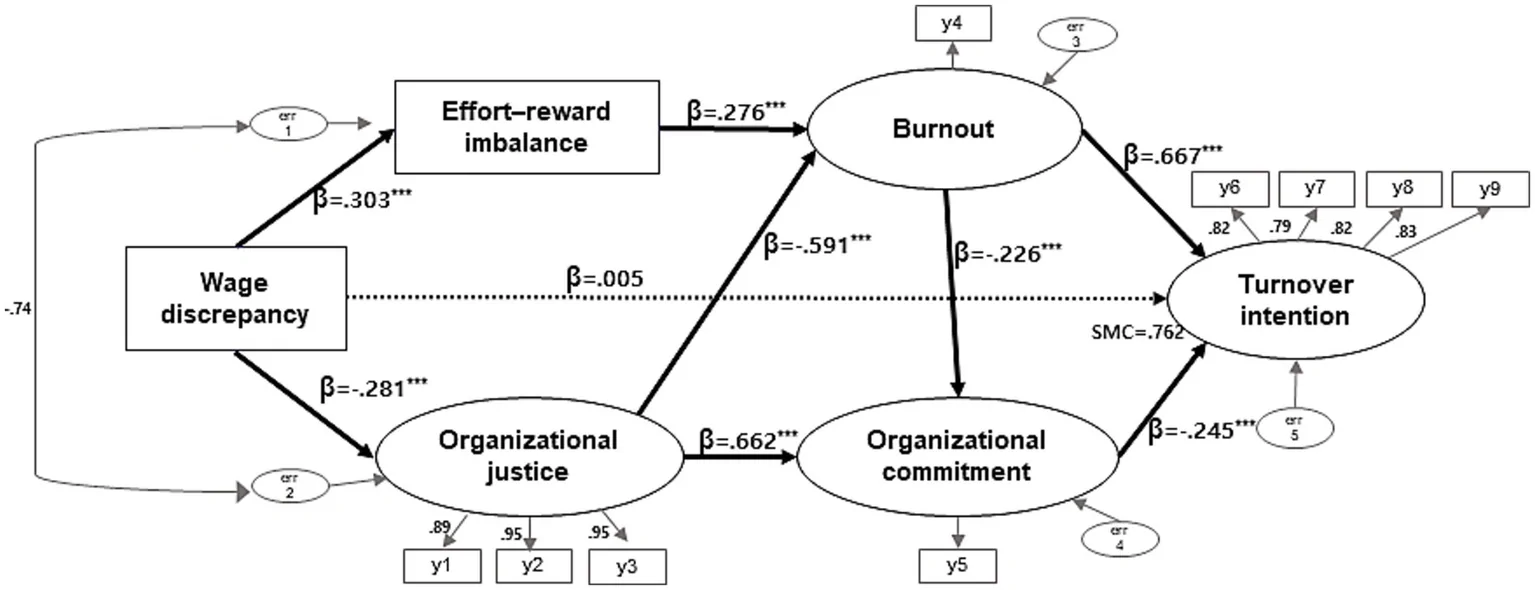
Hypothesized model for nurses’ turnover intention.

## 3 Methods

### 3.1 Study design

This cross-sectional descriptive survey examined the structural relationships among wage discrepancy, ERI, organizational justice, burnout, organizational commitment, and turnover intention among nurses working in healthcare institutions nationwide; the relationships were evaluated using structural equation modeling (SEM).

### 3.2 Participants

Eligible participants were nurses with at least 3 months of clinical experience who were employed in tertiary hospitals, general hospitals, or hospitals nationwide and agreed to participate in the study. Nurses working in clinics and nurse managers at or above the level of head nurse were excluded.

The target sample size was established prospectively based on the planned quota-stratified recruitment design rather than on a fixed sample-size rule for structural equation modeling. The sampling framework comprised six predefined strata formed by crossing two geographical regions (metropolitan and nonmetropolitan) with three hospital types (tertiary hospital, general hospital, and hospital). Approximately 100–150 eligible nurses were targeted within each of the six strata to ensure sufficient coverage of the predefined geographical and hospital-type groups, with an overall recruitment target of approximately 800 participants. Quota-based recruitment is commonly used to control sample composition across predefined subgroups when participants are recruited from online nonprobability panels (24). The quota-stratified recruitment strategy was intended to ensure coverage across the predefined geographical regions and hospital types and to reduce substantial imbalance in sample composition. Because participants were recruited from an existing online panel rather than randomly selected from a national sampling frame of hospital nurses, the resulting sample should not be considered nationally representative.

A total of 800 nurses completed the survey. After excluding eight responses that did not meet the predefined data-quality criteria because of incomplete or unreliable responses, 792 participants were included in the final analysis.

### 3.3 Measurements

For instruments that had been translated or adapted into Korean or shortened from the original versions, permission for use was obtained from the respective authors prior to data collection.

#### 3.3.1 Wage level discrepancy

Wage level discrepancy was defined as the difference between the current annual wage an individual receives and the annual wage perceived as appropriate. Previous research on pay-level satisfaction suggests that employees evaluate their wage not only in terms of the amount actually received but also in relation to the amount they perceive as appropriate (25). In this study, current annual wage and perceived appropriate annual wage were assessed using two items asking participants to report their current annual wage and the annual wage they considered appropriate, respectively. Wage level discrepancy was calculated as the ratio of perceived appropriate annual wage to current annual wage (perceived appropriate annual wage/current annual wage). A ratio of 1.00 indicates that the current annual wage is equal to the perceived appropriate annual wage. A ratio greater than 1.00 indicates that the perceived appropriate annual wage exceeds the current annual wage, whereas a ratio below 1.00 indicates that the current annual wage exceeds the perceived appropriate annual wage. Thus, among ratios greater than 1.00, higher values indicate a greater discrepancy between the current wage received and the wage perceived as appropriate by the participant. Accordingly, the wage discrepancy ratio primarily reflects the extent to which the current annual wage falls short of the participant’s perceived appropriate annual wage.

#### 3.3.2 Effort–reward imbalance

ERI refers to a lack of reciprocity in which the rewards employees receive, such as pay, recognition, job security, and career opportunities, are insufficient relative to the effort they invest in their work. This imbalance represents a form of psychosocial occupational stress that may lead to persistent stress responses and adverse health outcomes (14).

In this study, ERI was measured using the Effort–Reward Imbalance Short Form developed by Hwang et al. (26). The instrument consists of 14 items: three assessing effort, seven assessing reward, and four assessing overcommitment. Each item is rated on a 4-point Likert scale, ranging from 1 (strongly disagree) to 4 (strongly agree).

The ERI ratio was calculated by dividing the summed score of the effort subscale by the summed score of the reward subscale and multiplying the resulting value by a correction factor of 7/3 to account for the unequal numbers of items in the two subscales. An ERI ratio of ≥1 indicates that the rewards received are insufficient relative to the effort invested, reflecting a higher degree of ERI. Although overcommitment was measured as a component of the original instrument, it was not included in the hypothesized model and was, therefore, excluded from the SEM analysis.

In the study by Hwang et al. (26), Cronbach’s α coefficients were 0.75 for effort, 0.74 for reward, and 0.72 for overcommitment. In the present study, Cronbach’s α coefficients were 0.64 for effort and 0.79 for reward.

#### 3.3.3 Organizational justice

Organizational justice refers to the extent to which organizational members perceive organizational outcomes and the processes used to determine them as fair. It is divided into distributive justice, which concerns the principle that rewards should be distributed according to individual contributions; procedural justice, which concerns the perceived fairness of procedures and rules used in organizational decision-making; and interactional justice, which concerns perceptions of fairness in interpersonal treatment arising from communication between supervisors and subordinates within the organization (27). In this study, organizational justice was measured using the instrument developed by Niehoff and Moorman (27) and translated into Korean by Jung and Choi (28). The instrument consists of 20 items: five items on distributive justice, six items on procedural justice, and nine items on interactional justice. Each item was measured on a 7-point Likert scale: 1, strongly disagree; 2, generally disagree; 3, slightly disagree; 4, neutral; 5, slightly agree; 6, agree; and 7, strongly agree. Possible scores ranged from 5 to 35 for distributive justice, 6 to 42 for procedural justice, and 9 to 63 for interactional justice. Higher scores for each subdomain indicate that participants perceive a higher level of fairness in the corresponding organizational domain. At the time of instrument development, Niehoff and Moorman (27) reported Cronbach’s α values of 0.76 for distributive justice, 0.85 for procedural justice, and 0.92 for interactional justice; Jung and Choi (28) reported an overall value of 0.96; in this study, the corresponding value was 0.97.

#### 3.3.4 Burnout

Burnout was measured using an instrument originally developed by Pines et al. (29), which was revised by Choi (30). The instrument consists of 13 items across three subdomains: five physical items, five emotional items, and three mental items. Each item is rated on a 5-point Likert scale, ranging from 1, strongly disagree, to 5, strongly agree. Higher scores indicate a higher level of burnout. Cronbach’s α was 0.94 in the study by Choi (30), and it was 0.88 in the present study.

#### 3.3.5 Organizational commitment

Organizational commitment refers to a state in which organizational members accept the organization’s goals and, based on their attachment to the organization, are motivated to exert their best efforts on its behalf (31). In this study, organizational commitment was measured using nine items from the instrument developed by Mowday et al. (32), which was modified and supplemented by Noh and Jung (33) for use in nursing organizations. The items are rated on a 5-point Likert scale, ranging from 1, strongly disagree, to 5, strongly agree, with higher scores indicating a higher level of organizational commitment. Cronbach’s α was 0.89 in the study by Noh and Jung (33), and it was 0.92 in the present study.

#### 3.3.6 Turnover intention

Turnover intention refers to an individual’s intention to leave their current organization. In this study, turnover intention was measured using the instrument developed by Park (34), who adapted the four turnover-intention items used by Lawler (35) for application to nurses. The items are rated on a 5-point Likert scale, ranging from 1, strongly disagree, to 5, strongly agree, with higher scores indicating greater turnover intention. Cronbach’s α was 0.88 in the study by Park (34), and it was 0.89 in this study.

#### 3.3.7 General and work-related characteristics

The general and work-related characteristics assessed in this study were sex, age, marital status, educational level, position, work schedule, clinical experience, years in the current hospital, current department, hospital location, hospital type, number of beds, current annual wage, perceived appropriate annual wage, location of first employment after graduation, key factors in choosing the first hospital after graduation, effect of wage level on turnover intention, and turnover experience.

### 3.4 Data collection and ethical considerations

Ethical approval for this study was obtained from the Institutional Review Board of Gyeongsang National University prior to data collection (approval No. GIRB-A26-NY-0045). Data were collected from May to June 2026 through a professional online survey company’s nationwide registered research panel. The researcher provided the survey company with the predefined eligibility criteria and recruitment quotas for the six strata based on geographical region and hospital type. Recruitment continued within each stratum until the prespecified quota was reached. Eligible panel members who met the recruitment criteria received an invitation to participate through the survey company and accessed the survey via a secure online link. Before beginning the questionnaire, potential participants were provided with an online participant information sheet describing the study purpose, procedures, expected participation time, voluntary nature of participation, confidentiality of the collected information, and their right to decline or discontinue participation without disadvantage. Only participants who provided electronic informed consent could proceed to the questionnaire. Participants who completed the survey received a KRW 30,000 gift certificate as compensation for their participation.

### 3.5 Data analysis

Data were analyzed using IBM SPSS Statistics version 25.0 and IBM SPSS Amos version 25.0 (IBM Corp., Armonk, NY, USA). Participants’ general and work-related characteristics were summarized using frequencies, percentages, means, and standard deviations, as appropriate. The internal consistency of the multi-item scales was evaluated using Cronbach’s α coefficients, and normality was assessed using skewness and kurtosis. Pearson’s correlation coefficients were calculated to examine associations among the study variables, and multicollinearity was assessed using tolerance values and variance inflation factors (VIFs). Confirmatory factor analysis (CFA) was conducted for organizational justice and turnover intention, which were specified as latent constructs in the structural model. Organizational justice was represented by its three subdomains, whereas turnover intention was represented by its four individual items. Convergent validity was evaluated using standardized factor loadings, composite reliability (CR), and average variance extracted (AVE), and discriminant validity was assessed by comparing the AVE values with the squared correlation between the latent constructs. Wage discrepancy and effort–reward imbalance (ERI) were entered directly into the structural model as observed variables using their calculated scores. In contrast, burnout and organizational commitment were specified as single-indicator latent variables using their respective composite scale scores as indicators; for each construct, the factor loading was fixed at 1.0 and the measurement-error variance was fixed at 0. For organizational justice and turnover intention, which were specified as multiple-indicator latent constructs as described above, one factor loading was fixed at 1.0 to establish the measurement scale, while the remaining factor loadings and measurement-error variances were freely estimated. Geographic region (metropolitan vs. nonmetropolitan) was included as an observed control variable in the structural model. Structural equation modeling (SEM) was performed using maximum likelihood (ML) estimation. Overall model fit was evaluated collectively using the chi-squared statistic (χ^2^), normed chi-squared (χ^2^/df), standardized root mean square residual (SRMR), root mean square error of approximation (RMSEA), comparative fit index (CFI), and Tucker–Lewis index (TLI), rather than relying on a single fit index. Direct, indirect, and total effects were estimated, and indirect effects were tested using bias-corrected bootstrapping with 5,000 resamples and 95% confidence intervals. The hypothesized model was compared with a more parsimonious competing model in which the nonsignificant direct path from wage discrepancy to turnover intention was removed. Model comparison was based on the chi-squared difference test, Akaike information criterion (AIC), Bayesian information criterion (BIC), overall fit indices, and the stability of the remaining parameter estimates. The competing model was examined to determine whether removal of the theoretically specified direct path materially altered model fit or the substantive interpretation of the findings.

## 4 Results

### 4.1 Participants’ characteristics

In total, 792 nurses participated in this study. The mean age was 29.78 (SD = 4.09) years, and 99.1% were women. The participants were employed in tertiary hospitals (38.8%), general hospitals (36.1%), and hospitals (25.1%). Approximately half of the participants worked in metropolitan areas (50.5%). The mean current annual wage was KRW 46.92 million (SD = 10.30 million), whereas the mean perceived appropriate annual wage was KRW 53.50 million (SD = 12.66 million). Overall, 40.2% of the participants had previous turnover experience, and 75.4% perceived that wage level influenced their turnover intention (Table 1).

**Table 1:** General and work-related characteristics of the participants (*n* = 792).

Characteristics	Categories	*n*	%	Mean±SD	Range
Sex	F	785	99.1		
	M	7	0.9		
Age (years)	≤25	80	10.1	29.78 ± 4.09	23–52
	26–30	447	56.4		
	31–35	210	26.5		
	≥36	55	6.9		
Marital status	Single	639	80.7		
	Married	153	19.3		
Educational level	Associate degree	36	4.5		
	Bachelor’s degree	737	93.1		
	Master’s degree	19	2.4		
Position	Staff nurse	764	96.5		
	Charge nurse	28	3.5		
Work schedule	Fixed	151	19.1		
	Shift	641	80.9		
Clinical experience (years)	<3	103	13.0	5.63 ± 3.48	0.67–30.00
	3–<5	313	39.5		
	5–<7	190	24.0		
	≥7	186	23.5		
Years in current hospital	<3	264	33.3	4.39 ± 3.11	0.25–30.00
	3–<5	275	34.7		
	5–<7	165	20.8		
	≥7	88	11.1		
Current department	General ward	696	87.9		
	ICU/ER/OR	61	7.7		
	OPD, etc.	35	4.4		
Hospital location	Metropolitan	400	50.5		
	Nonmetropolitan	392	49.5		
Hospital type	Tertiary hospital	307	38.8		
	General hospital	286	36.1		
	Hospital	199	25.1		
Number of beds	<100	138	17.4	642.72 ± 601.85	30–5,000
	100–499	272	34.3		
	500–999	198	25.0		
	≥1,000	184	23.2		
Current annual wage (million KRW)	<40	196	24.7	46.92 ± 10.30	30–100
	40–49	298	37.6		
	50–59	220	27.8		
	≥60	78	9.8		
Perceived appropriate annual wage (million KRW)	<50	340	42.9	53.50 ± 12.66	34–150
	50–59	238	30.1		
	60–69	120	15.2		
	≥70	94	11.9		
Location of first employment after graduation	Metropolitan	400	50.5		
	Nonmetropolitan	392	49.5		
Key factors in choosing the first hospital after graduation	Compensation and employment conditions	377	47.6		
	Size and recognition of the hospital	139	17.6		
	Career development and growth opportunities	81	10.2		
	Working environment	81	10.2		
	Region and accessibility	72	9.1		
	Personal and social factors	40	5.1		
	Others	2	0.3		
Effect of wage level on turnover intention	Disagree	40	5.1		
	Neutral	155	19.6		
	Agree	597	75.4		
Turnover experience	Yes	318	40.2		
	No	474	59.8		

ER, emergency room; ICU, intensive care unit; OPD, outpatient department; OR, operating room.

### 4.2 Differences in current annual wage according to hospital region and type

After adjusting for clinical experience, nurses working in metropolitan-area hospitals had significantly higher current annual wages than nurses working in nonmetropolitan hospitals (*F* = 4.70, *p* = 0.031). This regional difference remained significant in tertiary hospitals (*F* = 6.38, *p* = 0.012) and general hospitals (*F* = 15.62, *p* < 0.001) but not in smaller hospitals.

Current annual wage significantly differed according to hospital type (*F* = 333.54, *p* < 0.001). Analyses stratified by hospital location revealed significant differences in annual wage across hospital types in both metropolitan areas (*F* = 101.11, *p* < 0.001) and nonmetropolitan areas (*F* = 382.25, *p* < 0.001), with tertiary hospitals reporting the highest wages, followed by general hospitals and hospitals (Table 2).

**Table 2:** Differences in current annual wages according to hospital location and type (*n* = 792).

Variable	Category	Geographic region		Total, Mean± SD	*F*	*p*
		Mean± SD				
		Metropolitan (*n* = 400)	Nonmetropolitan (*n* = 392)			
Hospital type	Tertiary^a^ (*n* = 307)	54.49 ± 12.31	52.90 ± 5.74	53.72 ± 9.71	6.38	0.012
	General^b^ (*n* = 286)	47.24 ± 11.72	42.50 ± 4.97	44.87 ± 9.29	15.62	<0.001
	Hospital^c^ (*n* = 199)	39.70 ± 5.10	39.06 ± 3.34	39.38 ± 4.31	1.47	0.227
Total		48.24 ± 12.20	45.58 ± 7.68	46.92 ± 10.30	4.70	0.031
*F*		101.11	382.25	333.54		
*p*		<0.001	<0.001	<0.001		
*Post-hoc* (Bonferroni)		a > b > c	a > b > c	a > b > c		

Values are presented as raw means ± standard deviations (SDs). Differences in current annual wage were tested after controlling for clinical experience. Wage is expressed in million KRW. a–c Superscript letters indicate the results of post-hoc comparisions; groups with different letters differ significantly (*p*<0.05).

### 4.3 Descriptive statistics and correlations

The mean wage discrepancy ratio was 1.14 (SD = 0.13), indicating that participants perceived an appropriate annual wage to be approximately 14% higher than their current annual wage. The mean ERI ratio was 1.22 (SD = 0.58). The mean organizational justice score was 4.24 (SD = 1.30) on a 7-point scale, with mean scores of 3.92 (SD = 1.39) for distributive justice, 4.36 (SD = 1.35) for procedural justice, and 4.33 (SD = 1.34) for interactional justice. The mean scores for burnout, organizational commitment, and turnover intention were 2.99 (SD = 0.88), 3.24 (SD = 0.92), and 3.21 (SD = 1.06), respectively, on a 5-point scale. As shown in Table 3, the study variables showed significant correlations in the expected directions. Wage discrepancy was positively correlated with ERI and burnout and negatively correlated with organizational justice and organizational commitment. ERI was negatively correlated with organizational justice and organizational commitment and positively correlated with burnout and turnover intention. Organizational justice was negatively correlated with burnout and turnover intention and positively correlated with organizational commitment. Burnout was negatively correlated with organizational commitment and positively correlated with turnover intention, whereas organizational commitment was negatively correlated with turnover intention. The absolute correlation coefficients ranged from 0.205 to 0.799 (all *p* < 0.05) (Table 3).

**Table 3:** Descriptive statistics of the variables, normality tests, and correlations.

Variable		Mean±SD	Skew-ness	Kurtosis	WD	ERI	OJ (total)	BO	OC	TI
					r(*p*)					
WD		1.14 ± 0.13	1.83	4.74	1					
ERI	Effort	2.87 ± 0.65			0.265 (<0.001)	1				
	Reward	2.62 ± 0.62								
	ERI ratio	1.22 ± 0.58	1.46	3.16						
OJ	Distributive justice	3.92 ± 1.39	0.21	−0.77	−0.205 (<0.001)	−0.750 (<0.001)	1			
	Procedural justice	4.36 ± 1.35	−0.40	−0.40						
	Interactional justice	4.33 ± 1.34	−0.29	−0.47						
	Total	4.24 ± 1.30	−0.20	−0.37						
BO		2.99 ± 0.88	−0.01	−0.70	0.321 (<0.001)	0.701 (<0.001)	−0.742 (<0.001)	1		
OC		3.24 ± 0.92	−0.54	−0.56	−0.308 (<0.001)	−0.641 (<0.001)	0.786 (<0.001)	−0.726 (<0.001)	1	
TI		3.21 ± 1.06	−0.59	−0.71	0.258 (<0.001)	0.564 (<0.001)	−0.664 (<0.001)	0.799 (<0.001)	−0.692 (<0.001)	1

BO, burnout; ERI, effort–reward imbalance; OC, organizational commitment; OJ, organizational justice; TI, turnover intention; WD, wage discrepancy.

### 4.4 Measurement model

#### 4.4.1 Confirmatory factor analysis

Confirmatory factor analysis (CFA) was conducted to evaluate the measurement model, in which organizational justice and turnover intention were specified as latent constructs. Organizational justice was represented by its three subdomains—distributive, procedural, and interactional justice—whereas turnover intention was represented by its four individual items. The standardized factor loadings ranged from 0.885 to 0.953 for organizational justice and from 0.796 to 0.830 for turnover intention. The composite reliability values were 0.909 for organizational justice and 0.840 for turnover intention, and the corresponding average variance extracted (AVE) values were 0.769 and 0.567, respectively, supporting convergent validity.

Discriminant validity was also supported. The correlation between organizational justice and turnover intention was −0.711, yielding a squared correlation of 0.506, which was lower than the AVE values for both organizational justice (0.769) and turnover intention (0.567).

The measurement model yielded χ^2^ = 189.63 (*df* = 33, *p* < 0.001), χ^2^/*df* = 5.75, SRMR = 0.029, RMSEA = 0.077, GFI = 0.957, AGFI = 0.915, NFI = 0.975, TLI = 0.965, and CFI = 0.979. Although the χ^2^/*df* value was elevated, the remaining fit indices collectively indicated acceptable overall fit of the measurement model.

#### 4.4.2 Assessment of normality and multicollinearity

The absolute values of skewness and kurtosis were below 2 and 7, respectively, indicating acceptable univariate normality. Tolerance values ranged from 0.141 to 0.848, and variance inflation factor (VIF) values ranged from 1.179 to 7.090, with no evidence of severe multicollinearity according to the prespecified criteria.

#### 4.4.3 Validation of the hypothesized model for nurses’ turnover intention

The fit indices provided mixed evidence regarding the overall fit of the hypothesized structural model: χ^2^ = 344.04, df = 44, *p* < 0.001, χ^2^/df = 7.82, SRMR = 0.043, RMSEA = 0.093, CFI = 0.961, and TLI = 0.942. Although the CFI and SRMR indicated favorable fit and the TLI was relatively close to commonly used criteria, the RMSEA and χ^2^/df indicated less-than-optimal fit. Therefore, the model fit was interpreted cautiously, based on the overall pattern of fit indices rather than any single index. Eight of the nine hypothesized structural paths were statistically significant. Wage discrepancy was positively associated with ERI (*β* = 0.303, *p* < 0.001) and negatively associated with organizational justice (*β* = −0.281, *p* < 0.001). ERI (*β* = 0.276, *p* < 0.001) and organizational justice (*β* = −0.591, *p* < 0.001) were significantly associated with burnout, together accounting for 63.8% of its variance. Organizational justice was positively associated with organizational commitment (*β* = 0.662, *p* < 0.001), whereas burnout was negatively associated with organizational commitment (*β* = −0.226, *p* < 0.001), together accounting for 69.1% of its variance. Burnout (*β* = 0.667, *p* < 0.001) and organizational commitment (*β* = −0.245, *p* < 0.001) together accounted for 76.2% of the variance in turnover intention. However, the direct path from wage discrepancy to turnover intention was not statistically significant (*β* = 0.005, *p* = 0.833) (Table 4; Figure 1).

**Table 4:** Comparison of parameter estimates between the hypothesized and competing models.

Model	Endogenous variables	Exogenous variables	B	S. E.	*β*	C. R.	*p*	SMC
Hypothesized	ERI	WD	1.359	0.149	0.303	9.113	<0.001	0.126
	OJ	WD	−2.782	0.335	−0.281	−8.318	<0.001	0.144
	BO	ERI	0.414	0.054	0.276	7.702	<0.001	0.638
		OJ	−0.401	0.026	−0.591	−15.667	<0.001	
	OC	OJ	0.470	0.026	0.662	18.240	<0.001	0.691
		BO	−0.237	0.035	−0.226	−6.693	<0.001	
	TI	WD	0.035	0.166	0.005	0.211	0.833	0.762
		BO	0.743	0.039	0.667	19.131	<0.001	
		OC	−0.261	0.034	−0.245	−7.679	<0.001	
Competing	ERI	WD	1.359	0.149	0.303	9.113	<0.001	0.126
	OJ	WD	−2.782	0.335	−0.281	−8.318	<0.001	0.144
	BO	ERI	0.414	0.054	0.276	7.702	<0.001	0.638
		OJ	−0.401	0.026	−0.591	−15.667	<0.001	
	OC	OJ	0.470	0.026	0.662	18.240	<0.001	0.691
		BO	−0.237	0.035	−0.226	−6.693	<0.001	
	TI	BO	0.744	0.039	0.668	19.247	<0.001	0.762
		OC	−0.261	0.034	−0.245	−7.735	<0.001	

BO, burnout; ERI, effort–reward imbalance; OC, organizational commitment; OJ, organizational justice; TI, turnover intention; WD, wage discrepancy.

B, unstandardized coefficient; *β*, standardized estimate; C. R., critical ratio; S. E., standard error; SMC, squared multiple correlation; representing the proportion of variance in each endogenous variable explained by all predictors specified in the structural model. For ERI and OJ, the SMC values reflect the combined explanatory contribution of wage discrepancy and geographic region.

#### 4.4.4 Direct, indirect, and total effects

Effect decomposition showed that wage discrepancy had no significant direct effect on turnover intention but had a significant indirect effect through ERI, organizational justice, burnout, and organizational commitment (*β* = 0.226, *p =* 0.001), resulting in a significant total effect (*β* = 0.231, *p =* 0.001). Burnout showed the strongest direct effect on turnover intention (*β* = 0.667, *p* < 0.001), whereas organizational commitment showed a significant negative direct effect (*β* = −0.245, *p* < 0.001). Organizational justice and ERI were associated with turnover intention only indirectly through burnout and organizational commitment (Table 5).

**Table 5:** Direct, indirect, and total effects in the hypothesized and competing models.

Model	Endogenous variables	Exogenous variables	Direct effect (*p*)	Indirect effect (*p*)	95% Bootstrap CI [LL-UL]	Total effect (*p*)
Hypothesized	ERI	WD	0.303 (<0.001)	-		0.303 (<0.001)
	OJ	WD	−0.281 (<0.001)	-		−0.281 (<0.001)
	BO	WD	-	0.249 (<0.001)	[0.195, 0.303]	0.249 (<0.001)
		ERI	0.276 (<0.001)	-		0.276 (<0.001)
		OJ	−0.591 (<0.001)	-		−0.591 (<0.001)
	OC	WD	-	−0.242 (<0.001)	[−0.300, −0.188]	−0.242 (<0.001)
		ERI	-	−0.062 (<0.001)	[−0.105, −0.031]	−0.062 (<0.001)
		OJ	0.662 (<0.001)	0.134 (<0.001)	[0.076, 0.197]	0.796 (<0.001)
		BO	−0.226 (<0.001)	-		−0.226 (<0.001)
	TI	WD	0.005 (0.833)	0.226 (0.001)	[0.176, 0.279]	0.231 (0.001)
		ERI	-	0.199 (<0.001)	[128, 0.271]	0.199 (<0.001)
		OJ	-	−0.589 (<0.001)	[−0.670, −0.506]	−0.589 (<0.001)
		BO	0.667 (<0.001)	0.055 (<0.001)	[0.030, 0.089]	0.722 (<0.001)
		OC	−0.245 (<0.001)	-		−0.245 (<0.001)
Competing	ERI	WD	0.303 (<0.001)	-		0.303 (<0.001)
	OJ	WD	−0.281 (<0.001)	-		−0.281 (<0.001)
	BO	WD	-	0.249 (<0.001)	[0.195, 0.303]	0.249 (<0.001)
		ERI	0.276 (<0.001)	-		0.276 (<0.001)
		OJ	−0.591 (<0.001)	-		−0.591 (<0.001)
	OC	WD	-	−0.242 (<0.001)	[−0.300, −0.188]	−0.242 (<0.001)
		ERI	-	−0.062 (<0.001)	[−0.105, −0.031]	−0.062 (<0.001)
		OJ	0.662 (<0.001)	0.134 (<0.001)	[0.076, 0.197]	0.796 (<0.001)
		BO	−0.226 (<0.001)	-	-	−0.226 (<0.001)
	TI	WD	-	0.226 (0.001)	[0.177, 0.275]	0.226 (0.001)
		ERI	-	0.200 (0.001)	[0.127, 0.270]	0.200 (0.001)
		OJ	-	−0.590 (<0.001)	[−0.669, −0.508]	−0.590 (<0.001)
		BO	0.668 (<0.001)	0.056 (<0.001)	[0.030, 0.089]	0.724 (<0.001)
		OC	−0.245 (<0.001)	-	-	−0.245 (<0.001)

BO, burnout; ERI, effort–reward imbalance; OC, organizational commitment; OJ, organizational justice; TI, turnover intention; WD, wage discrepancy. LL, lower limit; UL, upper limit; CI, confidence interval.

Indirect effects were tested using bootstrapping with 5,000 resamples. The 95% bootstrap confidence interval is reported for indirect effects; an indirect effect was considered statistically significant when the confidence interval did not include zero.

### 4.5 Competing model

The competing model, in which the nonsignificant direct path from wage discrepancy to turnover intention was removed, yielded lower AIC and BIC values than the hypothesized model (AIC = 410.08 vs. 412.04; BIC = 564.34 vs. 570.97; ΔAIC = 1.96, ΔBIC = 6.63). The chi-squared difference test indicated no significant difference in fit between the two models (Δχ^2^[1] = 0.04, *p* = 0.841). The path coefficients and indirect-effect estimates for ERI, organizational justice, burnout, and organizational commitment were nearly identical across the two models (Tables 4, 5). The hypothesized model, which included the *a priori* direct path from wage discrepancy to turnover intention, was retained as the primary model for reporting; the competing model was presented as a nested alternative used to assess the robustness of the indirect-effect estimates to the exclusion of the nonsignificant direct path (Figure 2).

**Figure 2:**
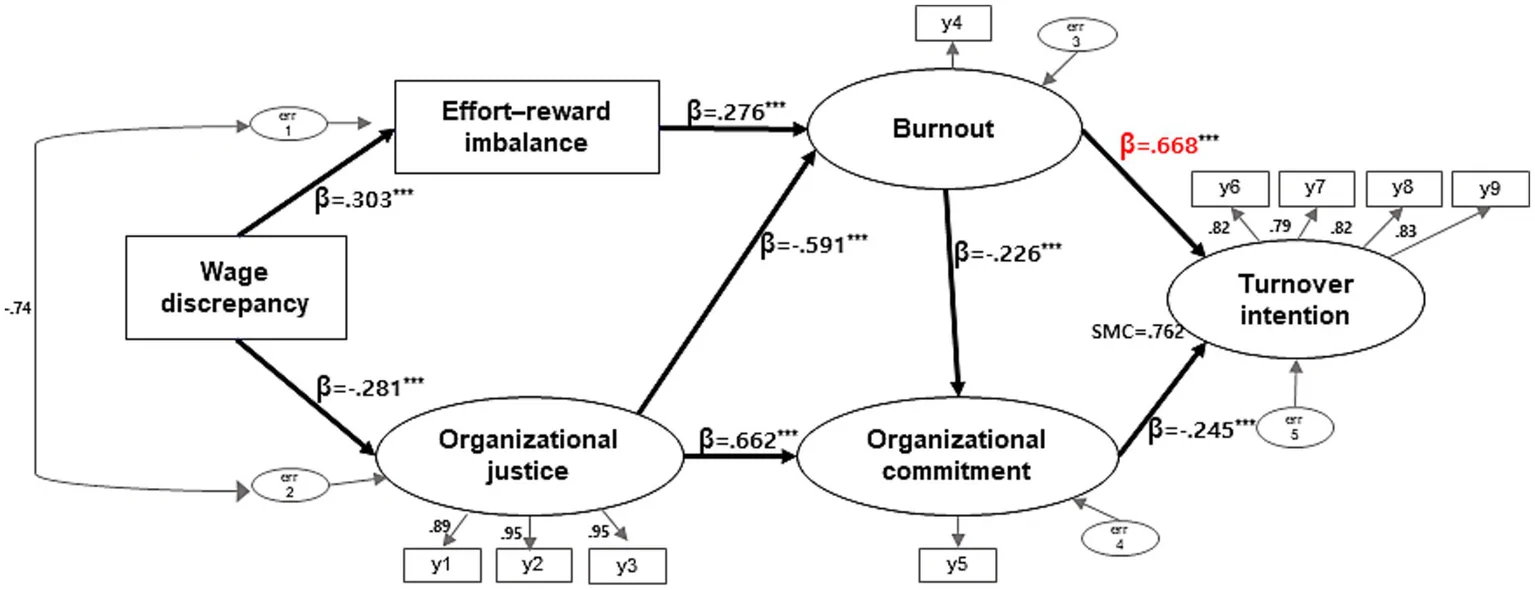
Competing model.

## 5 Discussion

The present study examined the structural associations linking wage discrepancy with turnover intention among hospital nurses. Three findings warrant emphasis. First, nurses reported a meaningful discrepancy between their current and perceived appropriate annual wages. Second, wage discrepancy was not directly associated with turnover intention but showed significant indirect associations through ERI and organizational justice. Third, burnout and organizational commitment were more proximal correlates of turnover intention, with burnout showing the strongest direct association. Taken together, these findings suggest that the association between wage discrepancy and turnover intention may be better understood within an integrated framework that considers nurses’ evaluations of effort–reward reciprocity and organizational fairness, together with burnout and organizational commitment. Rather than focusing solely on whether wage levels are associated with turnover intention, this study highlights how wage discrepancy is linked to turnover intention through a set of interrelated psychological and organizational factors.

First, the mean wage discrepancy ratio was 1.14, indicating that nurses, on average, considered an annual wage approximately 14% higher than their current wage to be appropriate. The mean current annual wage was 46.92 million KRW. The raw mean annual wage was 48.24 million KRW among nurses in metropolitan areas and 45.58 million KRW among those in nonmetropolitan areas. After controlling for clinical experience, the difference in current annual wage between the two geographic regions was statistically significant. The raw mean annual wage was also highest among nurses working in tertiary hospitals, followed by those in general hospitals and hospitals, with significant differences across hospital types after controlling for clinical experience. Descriptively, the highest raw mean annual wage was observed among nurses working in tertiary hospitals in metropolitan areas (54.49 million KRW), whereas the lowest was observed among those working in hospitals in nonmetropolitan areas (39.06 million KRW).

These findings are consistent with previous Korean studies documenting persistent wage disparities according to hospital characteristics and geographical location. In a nationwide survey of 3,742 hospital nurses in South Korea, Kim et al. (9) identified clinical experience, hospital ownership, and number of beds as significant determinants of nurses’ wages, whereas more recent evidence has demonstrated higher wages in metropolitan areas and an association between regional wage disparities and nurse staffing levels. Thus, although Korean nurses’ overall wage levels appear to have increased compared with earlier reports, substantial disparities according to hospital type and geographical location remain. These regional differences, together with the pronounced disparity between tertiary hospitals in metropolitan areas and hospitals in nonmetropolitan areas, suggest that geographical location and hospital type may jointly create economic incentives for nurses to move toward larger metropolitan hospitals, potentially contributing to persistent recruitment and retention difficulties in smaller and nonmetropolitan institutions.

Greater wage discrepancy was positively associated with ERI (*β* = 0.303) and negatively associated with organizational justice (*β* = −0.281). Although wage discrepancy had no significant direct effect on turnover intention, its indirect and total effects were significant, suggesting that its association with turnover intention may operate primarily through nurses’ perceptions of ERI and organizational justice. From the perspective of ERI theory, nurses who perceive their wages as inadequate relative to their workload, responsibilities, experiences, and professional expertise may be more likely to perceive that their rewards are not commensurate with their efforts (14). This interpretation is supported by previous evidence linking inadequate rewards, including salary, with greater ERI among nurses (36). Thus, wage discrepancy may be associated with nurses’ perceptions of imbalance between occupational effort and reward.

Wage discrepancy was also negatively associated with organizational justice, suggesting that perceived wage inadequacy may be associated not only with dissatisfaction with the amount of compensation but also with broader perceptions of organizational fairness. Employees evaluate not only whether the outcomes they receive are equitable but also whether the procedures used to allocate those outcomes and their interpersonal treatment are fair. Previous evidence of an association between organizational justice and turnover intention among healthcare workers supports the importance of such fairness perceptions (37). Notably, distributive justice had the lowest mean score among the three dimensions of organizational justice in the present study, suggesting that the fairness of reward allocation may be particularly salient to nurses.

These findings may also help explain inconsistencies in previous evidence regarding wages and nurse turnover. Higher wage levels have been associated with lower turnover intention, and wage level and hospital size have been identified as predictors of nurses’ actual turnover (11, 12). However, the association between wage level and turnover intention may be attenuated after job-related and organizational factors are considered (18). Together with the nonsignificant direct path identified in the present study, these findings suggest that compensation may be related to turnover intention not only through its monetary value but also through how nurses evaluate compensation relative to their contributions and the fairness of organizational reward systems. Accordingly, compensation strategies should consider not only wage levels but also transparent and equitable criteria reflecting workload, clinical responsibility, experience, and professional expertise.

ERI was positively associated with burnout (*β* = 0.276), whereas organizational justice was negatively associated with burnout (*β* = −0.591). A recent systematic review and meta-analysis estimated that more than half of nurses experienced ERI, with its prevalence increasing in more recent studies (38), underscoring the continuing relevance of ERI as a psychosocial work stressor in nursing. Consistent with the present findings, ERI is associated with greater burnout or emotional exhaustion among nurses (19, 20), suggesting that sustained high effort without commensurate rewards may deplete nurses’ emotional and psychological resources. Conversely, the negative association between organizational justice and burnout is consistent with previous findings among hospital nurses (21), suggesting that fair reward allocation, decision-making procedures, and interpersonal treatment may serve as organizational resources associated with lower burnout.

Notably, organizational justice showed a stronger association with burnout (*β* = −0.591) than did ERI (*β* = 0.276), suggesting that burnout was related both to whether nurses perceived rewards as commensurate with their efforts and, potentially more strongly, to how fairly they perceived their organizations to operate. In turn, burnout showed the strongest association with turnover intention (*β* = 0.667). This pattern is consistent with the proposed indirect pathway in which ERI is associated with turnover intention through burnout. Previous studies have similarly demonstrated associations between ERI and burnout and between burnout and turnover intention among nurses (19, 20, 39, 40). These findings suggest that perceived inadequate rewards and organizational unfairness are associated with burnout, which is in turn closely related to turnover intention. Therefore, improving compensation alone may be insufficient; retention strategies should also address organizational conditions associated with burnout, including workload and reward allocation, decision-making processes, work schedules, leadership support, and opportunities for adequate rest and recovery.

Organizational justice was positively associated with organizational commitment (*β* = 0.662), whereas burnout was negatively associated with organizational commitment (*β* = −0.226). Organizational commitment, in turn, was negatively associated with turnover intention (*β* = −0.245). The positive association between organizational justice and organizational commitment was consistent with previous findings among nurses (28, 40), supporting the present pathway. This pattern suggests that fair organizational practices may be related to greater trust, a stronger sense of belonging, and greater psychological attachment to the organization, all of which may be associated with stronger organizational commitment.

Similarly, the negative association between burnout and organizational commitment and the negative association between organizational commitment and turnover intention were consistent with previous findings among nurses (39–41), further supporting the pathways identified in the present model. These findings suggest that retention strategies should address not only factors associated with nurses’ intentions to leave, such as burnout and perceptions of inadequate or unfair rewards, but also factors that may strengthen retention. Organizational commitment may therefore represent an important retention-related factor. Healthcare organizations may support retention by recognizing professional contributions, providing career development opportunities, and fostering supportive environments in which nurses feel valued and connected to their organizations.

These pathway interpretations warrant caution because all variables were measured concurrently. The observed associations do not establish temporal ordering; for example, nurses with higher burnout or stronger turnover intention may also perceive their current wages as less adequate or their organizations as less fair. Beyond these individual-level associations, the observed differences in wages across regions and hospital types also have broader implications for the nursing workforce.

Nurses in metropolitan areas earned significantly higher wages than those in nonmetropolitan areas, and wages were highest in tertiary hospitals, followed by general hospitals and hospitals. These findings are particularly noteworthy because 75.4% of the participants perceived wage level as influencing turnover intention, and low salary or inadequate compensation was the most frequently reported reason for considering turnover (30.9%). Thus, movement from smaller or nonmetropolitan hospitals toward larger metropolitan institutions may reflect not only individual career preferences but also structural economic differences across regions and hospital types.

These findings have particular implications for smaller and nonmetropolitan hospitals, which may have limited financial capacity to compete with larger metropolitan institutions for experienced nurses. Therefore, policies aimed at reducing regional and institutional nursing workforce disparities should consider financial and workforce support for nonmetropolitan healthcare institutions, measures to narrow compensation gaps, and strategies to strengthen the wage competitiveness of smaller hospitals. Such policies should be complemented by career development opportunities, incentives for working in underserved regions, and improvements in working conditions. Addressing compensation adequacy together with organizational justice, burnout, and organizational commitment may ultimately contribute to improved nurse retention and a more equitable distribution of the nursing workforce.

The hypothesized model accounted for 76.2% of the variance in turnover intention in the present sample. However, this finding should be interpreted cautiously because the overall model-fit indices provided mixed evidence: whereas the CFI and SRMR indicated relatively favorable fit and the TLI was relatively close to commonly used criteria, the RMSEA and χ^2^/df indicated less-than-optimal fit. Moreover, the proportion of variance accounted for is an in-sample statistic from a cross-sectional model and should not be interpreted as evidence of strong predictive performance or causal explanatory power.

The more parsimonious competing model, which excluded the nonsignificant direct path from wage discrepancy to turnover intention, yielded slightly lower AIC and BIC values, but the chi-squared difference test was not significant and the remaining path estimates were essentially unchanged. The hypothesized model was therefore retained as the primary prespecified model to allow explicit evaluation of both the direct and indirect pathways. This decision should not be interpreted as evidence of superior empirical fit of the hypothesized model. Rather, the substantive interpretation was consistent across both model specifications: wage discrepancy showed no statistically significant direct association with turnover intention, whereas its association with turnover intention was represented primarily by indirect pathways involving ERI, organizational justice, burnout, and organizational commitment.

### 5.1 Strengths and limitations

This study has several strengths. It included a relatively large nationwide sample of nurses from different geographical regions and hospital types and examined wage discrepancy by considering both current and perceived appropriate wages rather than relying solely on absolute wage levels. Furthermore, integrating wage discrepancy, ERI, organizational justice, burnout, organizational commitment, and turnover intention within a single structural model enabled simultaneous examination of the pathways linking perceived wage inadequacy with turnover intention.

Several limitations should be considered. First, wage discrepancy was derived from nurses’ self-reported current wages and their perceptions of an appropriate wage. This measure was intended to capture perceived compensation adequacy, which was central to the study framework. However, perceptions of an appropriate wage may vary according to individual and contextual factors; therefore, wage discrepancy should be interpreted as a measure of perceived wage inadequacy rather than as an objective estimate of underpayment. Second, participants were recruited from a nationwide nonprobability online panel using quotas based on geographical region and hospital type. This approach may limit generalizability and introduce self-selection bias. Because recruitment occurred at the individual level, potential clustering of participants within hospitals was not explicitly modeled. Future studies using probability-based sampling and multilevel analytic approaches are warranted. Third, although the proposed model accounted for a substantial proportion of the variance in turnover intention, other relevant individual, work, and organizational factors were not included, and alternative pathways cannot be excluded. Finally, the cross-sectional design precludes establishing temporal ordering or causal direction among the observed associations. Although the proposed pathways were specified *a priori* on theoretical grounds, reverse or reciprocal relationships remain possible. For example, nurses experiencing greater burnout may perceive greater effort–reward imbalance or lower organizational justice, while nurses already considering leaving may report lower organizational commitment. In addition, turnover intention rather than actual turnover was examined as the outcome; therefore, the findings should not be interpreted as evidence that the identified pathways predict subsequent turnover behavior. Longitudinal studies incorporating repeated measurements, objective wage and employment data, actual turnover outcomes, and relevant individual- and organizational-level covariates are needed to clarify temporal ordering, examine reciprocal relationships, and test the proposed pathways more rigorously.

## 6 Conclusion

Wage discrepancy was associated with nurses’ turnover intention primarily through indirect organizational and psychological pathways in the proposed model rather than through a significant direct pathway. Perceived wage inadequacy was associated with greater ERI and lower organizational justice, and these factors were, in turn, associated with turnover intention through their relationships with burnout and organizational commitment. These findings extend the understanding of nurse turnover by highlighting that compensation-related perceptions, including the perceived adequacy and fairness of rewards relative to nurses’ contributions, are closely linked to organizational and psychological factors associated with turnover intention. Therefore, nurse retention policies should consider equitable compensation and organizational conditions that reduce burnout and strengthen organizational commitment.

## Data Availability

The datasets presented in this article are not readily available because the data supporting the findings of this study were generated by the authors through a nationwide survey of hospital nurses in South Korea. The dataset is not publicly available due to ethical and privacy considerations related to participant data. Requests to access the datasets should be directed to yumi825@gnu.ac.kr.
