# Supplementary material for: A structural pathway from wage discrepancy to turnover intention among hospital nurses: the mediating roles of effort–reward imbalance, organizational justice, burnout, and organizational commitment
Source: Front Public Health. 2026 Oct 20;14:1966344. doi: 10.3389/fpubh.2026.1966344 (PMC13727590; doi:10.3389/fpubh.2026.1966344)
Supplement: Supplementary file 1 [file Table_1.DOCX]

| Supplementary Table S1. Parameter estimates of the hypothesized and competing models including geographic region as a control variable (n=792) | | | | | | | | |
| --- | --- | --- | --- | --- | --- | --- | --- | --- |
| Model | Endogenous variables | Exogenous variables | B | S.E. | β | C.R. | *p* | SMC |
| Hypothesized | ERI | WD | 1.359 | 0.149 | 0.303 | 9.113 | <.001 | 0.126 |
|  |  | Geographic region | 0.218 | 0.039 | 0.185 | 5.566 | <.001 |  |
|  | OJ | WD | -2.782 | 0.335 | -0.281 | -8.318 | <.001 | 0.144 |
|  |  | Geographic region | -0.66 | 0.088 | -0.255 | -7.529 | <.001 |  |
|  | BO | ERI | 0.414 | 0.054 | 0.276 | 7.702 | <.001 | 0.638 |
|  |  | OJ | -0.401 | 0.026 | -0.591 | -15.667 | <.001 |  |
|  |  | Geographic region | -0.29 | 0.04 | -0.165 | -7.277 | <.001 |  |
|  | OC | OJ | 0.47 | 0.026 | 0.662 | 18.24 | <.001 | 0.691 |
|  |  | BO | -0.237 | 0.035 | -0.226 | -6.693 | <.001 |  |
|  |  | Geographic region | 0.205 | 0.04 | 0.111 | 5.078 | <.001 |  |
|  | TI | WD | 0.035 | 0.166 | 0.005 | 0.211 | 0.833 | 0.762 |
|  |  | BO | 0.743 | 0.039 | 0.667 | 19.131 | <.001 |  |
|  |  | OC | -0.261 | 0.034 | -0.245 | -7.679 | <.001 |  |
|  |  | Geographic region | 0.185 | 0.042 | 0.094 | 4.378 | <.001 |  |
| Competing | ERI | WD | 1.359 | 0.149 | 0.303 | 9.113 | <.001 | 0.126 |
|  |  | Geographic region | 0.218 | 0.039 | 0.185 | 5.566 | <.001 |  |
|  | OJ | WD | -2.782 | 0.335 | -0.281 | -8.318 | <.001 | 0.144 |
|  |  | Geographic region | -0.66 | 0.088 | -0.255 | -7.529 | <.001 |  |
|  | BO | ERI | 0.414 | 0.054 | 0.276 | 7.702 | <.001 | 0.638 |
|  |  | OJ | -0.401 | 0.026 | -0.591 | -15.667 | <.001 |  |
|  |  | Geographic region | -0.29 | 0.04 | -0.165 | -7.277 | <.001 |  |
|  | OC | OJ | 0.47 | 0.026 | 0.662 | 18.24 | <.001 | 0.691 |
|  |  | BO | -0.237 | 0.035 | -0.226 | -6.693 | <.001 |  |
|  |  | Geographic region | 0.205 | 0.04 | 0.111 | 5.078 | <.001 |  |
|  | TI | BO | 0.744 | 0.039 | 0.668 | 19.247 | <.001 | 0.762 |
|  |  | OC | -0.261 | 0.034 | -0.245 | -7.735 | <.001 |  |
|  |  | Geographic region | 0.183 | 0.042 | 0.093 | 4.330 | <.001 |  |
| Note. BO, burnout; ERI, effort–reward imbalance; OC, organizational commitment; OJ, organizational justice; TI, turnover intention; WD, wage discrepancy. B = unstandardized coefficient; S.E. = standard error; β = standardized estimate; C.R. = critical ratio; SMC = squared multiple correlation. Geographic region was coded as 0 = metropolitan and 1 = nonmetropolitan. | | | | | | | | |
